# Investigation of model stacking for drug sensitivity prediction

**DOI:** 10.1186/s12859-018-2060-2

**Published:** 2018-03-21

**Authors:** Kevin Matlock, Carlos De Niz, Raziur Rahman, Souparno Ghosh, Ranadip Pal

**Affiliations:** 1 Department of Electrical and Computer Engineering, Texas Tech University, 1012 Boston Ave, Lubbock, 79409 TX USA; 2 Department of Mathematics and Statistics, Texas Tech University, 1108 Memorial Circle, Lubbock, 79409 TX USA

**Keywords:** Drug sensitivity prediction, Stacking, Bias

## Abstract

**Background:**

A significant problem in precision medicine is the prediction of drug sensitivity for individual cancer cell lines. Predictive models such as Random Forests have shown promising performance while predicting from individual genomic features such as gene expressions. However, accessibility of various other forms of data types including information on multiple tested drugs necessitates the examination of designing predictive models incorporating the various data types.

**Results:**

We explore the predictive performance of model stacking and the effect of stacking on the predictive bias and squared error. In addition we discuss the analytical underpinnings supporting the advantages of stacking in reducing squared error and inherent bias of random forests in prediction of outliers. The framework is tested on a setup including gene expression, drug target, physical properties and drug response information for a set of drugs and cell lines.

**Conclusion:**

The performance of individual and stacked models are compared. We note that stacking models built on two heterogeneous datasets provide superior performance to stacking different models built on the same dataset. It is also noted that stacking provides a noticeable reduction in the bias of our predictors when the dominant eigenvalue of the principle axis of variation in the residuals is significantly higher than the remaining eigenvalues.

## Background

In precision medicine, drug sensitivity prediction is a significant problem. The primary goal of improving prediction accuracy for precision medicine opens up problems that are broadly relevant to other machine learning tasks. In this article, we examine the stacking of predictive models and their influence on prediction accuracy and modeling bias. The principal individual model considered in this article is Random Forests (RF) since previously reported studies [1–4] have shown RF to outperform multiple other approaches in drug sensitivity prediction applications. However, RF models can suffer from inherent bias where they under predict sensitivities above the mean and over predict sensitivities below the mean. There have been some recent studies to address this bias [5, 6] but none have explored the effect of stacking on bias. In this article, we illustrate that stacking of model predictions automatically lower the inherent bias in RF based models without having to resort to explicit bias reduction approaches. Furthermore, we explored the theoretical underpinnings of the stacking operation on mean squared error and how stacking will produce results that are no worse than the worst individual model.

To demonstrate the role of stacking in accuracy and bias reduction, we created a drug sensitivity prediction setup with multiple data sources. The main motivation behind stacking is that each model will provide complementary information. For that reason we have included a variety of different datasets to built our individual models. We consider multiple cell lines and multiple tested drugs as well as the genomic information in the form of gene expressions for each cell line. Each drug is characterized by its physical properties and its potential targets. The drug responses are normalized Area Under the curve (AUC) values obtained from cell viability curves. This setup allows us to explore incorporating complementary information in our prediction models. For instance, gene expression provides information on each cell line whereas drug targets provide information on each drug that is complementary to genomic information. Thus, the effect of both cell line and drug information can be included in prediction. Note that, we can train some models only on cell lines with fixed drug whereas other models can be trained on drugs with fixed cell line and they can be combined to produce an integrated prediction model. This study provides a theoretically sound, but easy to implement, methodology to jointly analyze multiple pharmacogenomics databases [7, 8] that include information on multiple cell lines and multiple drugs. Thus, for a new cancer patient, a biopsy can be used to generate a genomic profile of the patient and a drug screen can be run to get an estimate of the cell viability for the drugs in the screen and then we can utilize these information along with prior database information to predict sensitivities for drugs that have not been tested in the drug screen. Improvement in performance will motivate us to explore personalized medicine from the perspective of training using both genomic and drug specific features.

## Methods

### Drug sensitivity prediction

To investigate stacking performance, we selected individual modeling techniques that have previously shown to perform well for drug sensitivity predictions scenarios. These methods include Random Forest regression approach and Neural Network based prediction along with KNN based sensitivity estimation using drug target profiles. We provide a brief overview of these three approaches below.

#### Random forest

Random Forest regression refers to an ensemble of regression trees [9] where a set of *T* un-pruned regression trees are generated based on bootstrap sampling from the original training data. For selecting the feature for splitting at each node, a random set of *m* features from the total *M* features are used. The inclusion of the concepts of bagging (Bootstrap sampling for each tree) and random subspace sampling (node split selected from random subset of features) increase the independence of the generated trees. Thus the averaging of the prediction over multiple trees has lower variance compared to individual regression trees.

##### Process of splitting a node

Let *X*(*i*,*j*) and *Y*(*i*)(*i*=1,⋯,*n*;*j*=1,⋯,*M*) denote the training predictor features and output response samples respectively. At any node *η*_*P*_, we aim to select a feature *j*_*s*_ from a random set of *m* features and a threshold *z* to partition the node into two child nodes *η*_*L*_ (left node with samples satisfying *x*_*tr*_(*I*∈*η*_*P*_,*j*_*s*_)≤*z*) and *η*_*R*_ (right node with samples satisfying *X*(*i*∈*η*_*P*_,*j*_*s*_)>*z*). We consider the node cost as sum of square differences:

1 $$ D\left(\eta_{P}\right)=\sum_{i\in \eta_{P}}\left(Y(i)-\mu\left(\eta_{P}\right)\right)^{2}  $$

where *μ*(*η*_*P*_) is the expected value of *Y*(*i*) in node *η*_*P*_. Thus, the reduction in cost for partition *γ* at node *η*_*P*_ is

2 $$ C\left(\gamma, \eta_{P}\right) = D\left(\eta_{P}\right)-D\left(\eta_{L}\right)-D\left(\eta_{R}\right)  $$

The partition *γ*∗ that maximizes *C*(*γ*,*η*_*P*_) for all possible partitions is selected for node *η*_*P*_. Note that for a continuous feature with *n* samples, a total of *n* partitions needs to be checked. Thus, the computational complexity of each node split is *O*(*mn*). During the tree generation process, a node with less than *n*_*size*_ training samples is not partitioned any further.

##### Forest prediction

Using the randomized feature selection process, we fit the tree based on the bootstrap sample {(**X**_1_,*Y*_1_),...,(**X**_*n*_,*Y*_*n*_)} generated from the training data.

Let us consider the prediction based on a test sample **x** for the tree *Θ*. Let *η*(**x**,*Θ*) be the partition containing **x**, the tree response takes the form [9–11]:

3 $$ {Y}(\mathbf{x},\Theta) = \sum\limits_{i=1}^{n} w_{i}(\mathbf{x},\Theta) y(i)  $$

where the weights *w*_*i*_(**x**,*Θ*) are given by

4 $$ w_{i}\left(\mathbf{x},\Theta \right) = \frac{\mathbf{1}_{\left\{ \mathbf{x}_{tr}(i)\in\eta\left(\mathbf{x},\Theta \right) \right\} }} {\# \left\{r: \mathbf{x}_{tr}(i)\in\eta \left(\mathbf{x}_{tr}(r),\Theta \right)\right\}}  $$

Let the *T* trees of the Random forest be denoted by *Θ*_1_,⋯,*Θ*_*T*_ and let *w*_*i*_(**x**) denote the average weights over the forest i.e.

5 $$ w_{i}(\mathbf{x}) = \frac{1}{T}\sum\limits_{j=1}^{T} w_{i}\left(\mathbf{x},\Theta_{j}\right).   $$

The Random Forest prediction for the test sample **x** is then given by

6 $$ \overline{Y}(\mathbf{x}) = \sum_{i=1}^{n} w_{i}(\mathbf{x}) y(i)  $$

#### Neural networks

Deep Learning (DL) is a revived Neural Networks (NN) based approach that is increasingly becoming popular due to its high predictive power for scenarios with large number of samples.

**Fig. 1:**
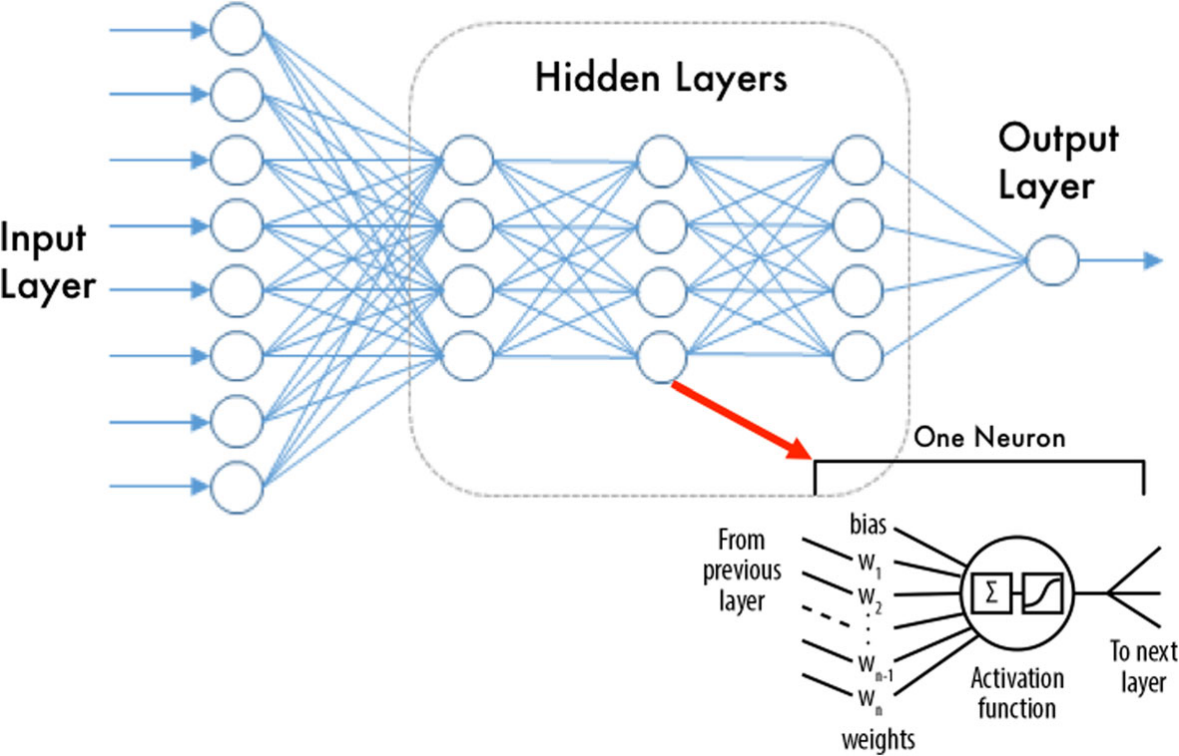
Deep Network layers and neuron details. Image generated from [14]

There exists a vast number of software options available to process a Deep Learning problem and we utilized the tool H2O [12], in its R-Package form. H2O is Java based, open source, multi-interface and multi-language machine learning and analytics platform that allows machine learning modeling using several algorithms including Deep Learning Neural Networks [13]. H2O deep learning module is based on a multi-layer feed-forward artificial neural network, trained with stochastic gradient descent (loss function minimization) using back-propagation [14]. An illustration of the neural network model is given in Fig. 1.

For this study the model parameters were selected after using grid search on a validation set. In general terms and for all the models, the early stopping deviance criteria was set to 0.001, with a *ℓ*_2_ regularization of 0.0001. The activation function chosen was a *Tanh* and 4 hidden layers with the same number of neurons for each layer. The number of neurons in each layer was set to be equal to the number of input features.

#### Sensitivity estimation using drug targets

Drug targets have been shown to be an effective source of data for estimating drug sensitivities [15]. However target data tends to be very sparse which limits the number of available methods. The k-Nearest Neighbor (KNN) algorithm is a simple yet powerful method of nonlinear classification that is popular in machine learning for sparse data. Given a set of training vectors *Φ* with their corresponding sensitivities *S*, we can estimate the sensitivity given a drug’s target profile by looking at the sensitivities corresponding to the k-closest training target profiles. For our case, we have chosen taxicab distance to pick the closest training samples, defined as:

$$d(\mathbf{\phi},\mathbf{\psi}) = \sum_{i=1}^{nTargets}|\phi_{i} - \psi_{i}| $$

The average of the *k* closest training vectors is our prediction. In our model we have chosen to look at *k*=5 closest samples.

We have developed two separate models for predicting drug sensitivity with KNN and target data. In the first method, which we denote as KNN Direct, we simply directly estimate AUC using all available target data for a single cell line. For the second method, called KNN Residual, we instead predict the residuals (actual values minus the mean) of each drug for a given cell line. We then add our residual prediction back onto the mean AUC of each drug for our final prediction.

### Integrated prediction

Up to this point, we have considered each model independently, i.e. one model for one set of data. However biological processes are complex and restricting our data to a single type rarely shows us the whole picture. To overcome this limitation, we have also utilized a systems genomics approach in our predictions.

From a machine learning point of view, we can format this as ensemble prediction problem. We first select *N*_*m*_ models, then let ${\boldsymbol {\tilde {y}}}=\left [y_{1},y_{2},...,y_{N_{m}}\right ]$ be the output of each of our individual models. The final prediction, *y*_*f*_ is formed using these individual predictions i.e.

$$y_{f} = \mathrm{C}({\boldsymbol{\tilde{y}}}) $$

#### Linear stacking

As the name implies, linear stacking is simply a linear combination of prediction algorithms i.e.

$$y_{f} = {\boldsymbol{\tilde{y}}}\,\mathbf{w} + b $$

where *w* is our set of linear weights for each model. We can easily solve for the weights utilizing matrix inverse to find the least squares solution.

Due to its high accuracy and low computational cost we have focused mainly on the Random Forest for our analysis of stacking. By comparison, the Neural Network has comparable accuracy but has significantly longer training train which did not make it practical for our purposes. It should be noted, however; that in principle linear stacking functions independent of the individual models and in most practical scenarios the model that has the highest accuracy for each given dataset should be chosen.

### Analysis of stacking

In this section we illustrate some attractive benefits of stacking operation besides being a simple tool to combine outputs from different models. Our main focus is on demonstrating how stacking reduces bias in Random Forest (RF) prediction. We conceptualize the distribution of ensemble predictions arising from each tree in the RF and frame a Bayesian Hierarchical model. It is shown that, under the assumption of Gaussianity, the Bayes rule, under mean square loss, turns out to be a linear combination of individual model outputs.

Denote the RF training dataset as $\mathcal {D}_{F}=(Y,\boldsymbol {x})$. We can view RF prediction as a weighted average of the individual tree prediction, i.e.,

7 $$ \bar{Y}(\boldsymbol{x})=\frac{1}{T}\sum_{j=1}^{T}\sum_{i=1}^{n} w_{i}\left(\boldsymbol{x},\Theta_{j}\right)y(i)  $$

Define the random variable $Z_{j}\left (\boldsymbol {x},\Theta _{j}\right)=\sum _{i=1}^{n} w_{i}\left (\boldsymbol {x},\Theta _{j}\right) y(i), \; j=1,2...,T$ as the prediction obtained from the *j*th tree generated by the *Θ*− process. Let us assume that the *Θ*− process induces a valid distribution on the finite collection

8 $$ \boldsymbol{Z}(\boldsymbol{x},\Theta)=\left[Z_{1}(\boldsymbol{x},\Theta_{1}),Z_{2}(\boldsymbol{x},\Theta_{2}),...,Z_{T}(\boldsymbol{x},\Theta_{T})\right]  $$

Now, observe that each tree attempts to predict the target *μ*(***x***)=*E*(*Y*|***x***) and the RF predictor, $\bar {Y}$ (obtained in 7), emerges as the sample average, $\bar {Z}$ of ***Z***(***x***). However, finite sample tree predictions are biased [6] resulting in *E*(*Z*_*j*_(***x***))=*α*_*j*_(*n*)+*β*_*j*_(*n*)*μ*(***x***) and $Var(Z_{j}(\boldsymbol {x}))=\sigma ^{2}_{j} \;\; j=1,2,..,T$, where the additive bias *α*_*j*_(*n*) is a sequence of constants that goes to 0 as *n*→*∞* and the multiplicative bias *β*_*j*_(*n*) is a sequence of constants that approaches 1 as *n*→*∞* under some smoothness condition on true *μ*(***x***) [16]. Note that, in this construction $\sigma ^{2}_{j}$ can be interpreted as the variance of individual tree estimates and, therefore, is of the order *k*_*n*_/*n* where *k*_*n*_ is approximately the number of terminal nodes and *n* is the number of samples on which the tree is built [17].

For illustration purpose, we assume *α*_*j*_=0, *β*_*j*_=*β*>0 and $\sigma ^{2}_{j}=\sigma ^{2},\;\; j=1,2,...,T$. In this formulation 0<*β*<1 is the event of underprediction by RF estimate, as is typical for small values of responses, and *β*>1 is the event of overprediction by RF estimate, as is typical for large values of responses [5]. For notational simplicity, we suppress the arguments *n*,*Θ* and ***x*** in relevant statistics henceforth. Under the assumption of Gaussianity and conditional independence, the joint distribution of [***Z***|*μ*,*β*,*σ*^2^] is $\prod _{j=1}^{T} { Normal}\left (\beta \mu,\sigma ^{2}\right)$. If there are no other models, we can assume a prior *π*(*μ*,*σ*^2^)∝1 (note that *μ* and *β* are not identifiable in this case) and the posterior mean of $\mu |\sigma ^{2},\mathcal {D}_{F}$ turns out to be the familiar RF estimate. Suppose, we have another model, *M*, potentially operating on a different set of inputs, ***x***_*m*_, but predicting the same response variable *Y*. We denote the training data for this model *M* as $\mathcal {D}_{M}$. The output of this model is *μ*_*m*_ which is an estimator of *E*(*Y*|***x***_*m*_). If we wish to pool both RF and model *M* together to generate predictions of *Y*, we can develop a hierarchical structure with *μ*_*m*_ as a prior mean for *μ*, so that the posterior of *μ* is conditional on both $\mathcal {D}_{F}$ and $\mathcal {D}_{M}$. For simplicity, let us assume conjugacy and impose a *Normal*(*μ*_*m*_,*τ*^2^) prior on *μ*. If *M* is another ensemble model, *τ*^2^ can be computed in the same vein as *σ*^2^. If *M* is deterministic, then a procedure to compute *τ*^2^ in a general setting is described in [18, 19].

Therefore the hierarchical specification takes the form

9 $$ \left[\boldsymbol{Z}|\mu,\sigma^{2},\beta\right]\left[\mu|\mu_{m},\tau^{2}\right]\left[\sigma^{2},\tau^{2},\beta\right]  $$

and the conditional posterior distribution of *μ*, $\left [\mu |\sigma ^{2},\tau ^{2},\beta,\mathcal {D}_{F},\mathcal {D}_{M}\right ]$, turns out to be Normal (*λ*,*ν*^2^), where,

10 $$\begin{array}{@{}rcl@{}} \nu^2&=&\left(\frac{1}{\tau^{2}}+\frac{T\beta^{2}}{\sigma^{2}}\right)^{-1}, \end{array} $$

11 $$\begin{array}{@{}rcl@{}} \lambda&=&\frac{T\beta\tau^{2}}{\sigma^2+T\beta^{2}\tau^{2}}\bar{Z}+\frac{\sigma^{2}}{\sigma^2+T\beta^{2}\tau^{2}}\mu_{m}. \end{array} $$

Note that, the Bayes estimate under square error loss is the posterior mean *λ* which happens to have similar form as the foregoing linear stacking estimator.

How is this representation of stacking estimator insightful? Observe that if *σ*^2^ is small, in particular if *σ*^2^≪*τ*^2^ and *β*>1 then $\lambda \approx \frac {1}{\beta }\bar {Z}$. Thus, when the ensembles in RF overpredicts, the stacking estimator downweighs the RF estimator (with negligible contribution from *μ*_*m*_) thereby reducing the bias. On the other hand, if *σ*^2^≪*τ*^2^ and 0<*β*<1 then $\lambda \approx \frac {C\beta }{1+C\beta ^{2}}\bar {Z}+\frac {1}{1+C\beta ^{2}}\mu _{m}$, where *C*=*Tτ*^2^/*σ*^2^≫1. In this situation RF ensemble underpredicts but stacking operation counteracts in the following way: (a) When *Cβ*≤1, the stacking estimate underweighs RF estimate but adds a non-trivial fraction of *μ*_*m*_. In an extreme situation, when $\beta (\in \mathbb {R}^{+})$ is in the neighborhood of 0, the stacking estimator does not put any weight on the RF estimate and solely uses *μ*_*m*_ as the prediction, thereby reducing the RF bias. (b) When *Cβ*≫1 the stacking estimator upweighs the RF estimate with minimal contribution from *μ*_*m*_. Clearly, in all the three foregoing situation, stacking helps reducing the bias of RF estimates.

What happens when *σ*^2^ and *τ*^2^ are comparable or *σ*^2^≫*τ*^2^? Our argument from previous paragraph suggests that the debiasing characteristic of stacking operation will critically hinge on *T*. However, arbitrarily large *T* is not useful because after a certain number of trees, individual tree outputs will be correlated hence violating the fundamental premise of conditional independence in our setup. Consequently, the effect of stacking operation on debiasing RF output is ambiguous.

Observe that in practise, we do not need to estimate the relevant parameters in the coefficient of $\bar {Z}$ and *μ*_*m*_ in (11). We can simply replace *μ* by observed responses (that are not used to obtain $\bar {Z}$ and *μ*_*m*_) and regress that on predictions obtained from RF and model *M*. The regression coefficients can be treated as the estimates of the coefficients in (11) while the intercept can be interpreted as an estimate of average additive bias. Thus, we argue that standard linear stacking operation should also behave according to the formulation above and will be an effective debiasing device subject to the variance condition.

The fact that we need *σ*^2^≪*τ*^2^ to force the stacking estimator operate as a debiasing devise indicates that we ought to design the stacking operation in such a way that the above condition is satisfied. Consider a generic situation where $\mathcal {D}_{M}$ consists of *n*_1_ independent samples and the feature matrix ***x***_*m*_ is of dimension *n*_1_×*p*_1_. $\mathcal {D}_{F}$ consists on *n*_2_ samples and the corresponding feature matrix ***x*** is of dimension *n*_2_×(*p*_1_+*p*_2_), with *col*(***x***_*m*_)⊂*col*(***x***), i.e., $\mathcal {D}_{F}$ includes all the features observed in $\mathcal {D}_{M}$ and also *p*_2_ additional feautes. We must predict the response utilizing the entire set of *p*_1_+*p*_2_ features. One can easily combine these two training sets by training an RF on $\mathcal {D}_{M}$, obtain *μ*_*m*_ and *τ*^2^ and then use this prior information on the RF trained on $\mathcal {D}_{F}$. In other words, one can simply stack RFs trained on $\mathcal {D}_{M}$ and $\mathcal {D}_{F}$. We call this operation *vertical stacking*. Since both are RF estimators *σ*^2^ is of order *k*_._/*n*_2_ and *τ*^2^ is of order *k*_._/*n*_1_. Since *k*_._ is typically user specifed and can be made to remain constant in both RFs, the variances of the stacking components are essentially determined by the sample sizes of the respective training set. Clearly, if *n*_2_<*n*_1_ the above condition relating the variances of the stacking components cannot be enforced. One can argue that the variance condition can be maintained by switching the generic label *σ*^2^ and *τ*^2^, but in this situation $\mathcal {D}_{F}$ contains more information as compared to $\mathcal {D}_{M}$ and hence we would like to put more weight on the RF trained on $\mathcal {D}_{F}$. In other words, the stacking operation should more effectively debias the RF estimates obtained from $\mathcal {D}_{F}$ than the other way around.

**Fig. 2:**
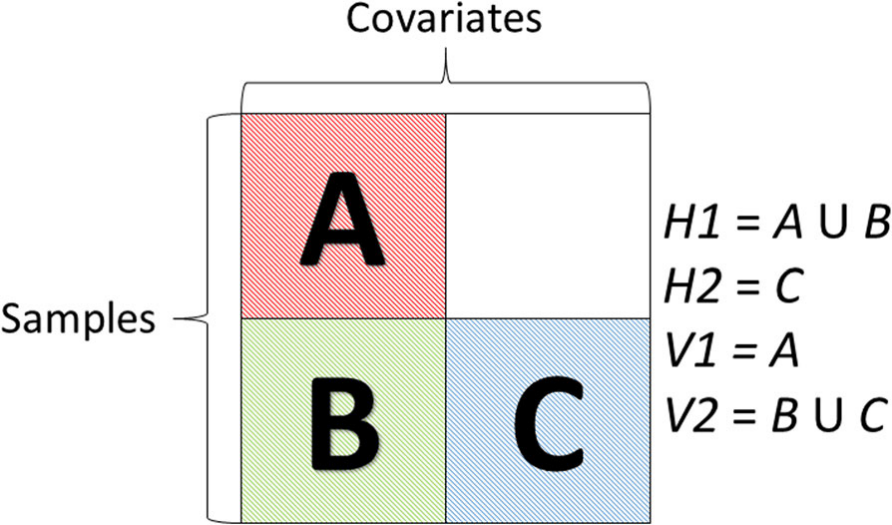
Stack Diagram

To enforce the variance condition, regardless of the sample sizes of $\mathcal {D}_{M}$ and $\mathcal {D}_{F}$, we introduce the notion of *horizontal stacking*. In this form of stacking we first partition the feature matrix associated with $\mathcal {D}_{F}$ into two parts $\boldsymbol {x}^{n_{2}\times (p_{1}+p_{2})}=\big (\boldsymbol {x}_{p_{1}}^{n_{2}\times p_{1}},\boldsymbol {x}_{p_{2}}^{n_{2}\times p_{2}}\big)$. We then train an RF on *n*_1_+*n*_2_ samples with feature matrix $\phantom {\dot {i}\!}(\boldsymbol {x}_{m},\boldsymbol {x}_{p_{1}})^{\prime }$. If *σ*^2^ is the variance associated with this stacking component, then *σ*^2^ is of the order *k*_._/(*n*_1_+*n*_2_). The other model is also an RF but trained on *n*_2_ samples and feature matrix $\boldsymbol {x}_{p_{2}}$. If *τ*^2^ is the variance associated with this stacking component, then *τ*^2^ is of the order *k*_._/*n*_2_. Keeping the number of terminal node constant, we can easily see *σ*^2^<*τ*^2^ and hence we expect *horizontal stacking* to be more efficient in debiasing than *vertical stacking*. Furthermore, as *n*_1_,*n*_2_→*∞* and *β*→1, it is easy to see, from (10), that the variance of horizontal stacking estimator is smaller than its vertical counterpart. Figure 2 provides a graphical representation of these two forms of stacking. Group *H*1 contains *n*_1_+*n*_2_ samples with *p*_1_ features while *H*2 contains only *n*_2_ samples with feature set *p*_2_. Our horizontal stacking predictor *Hc* is then the linear ensemble of *H*1 and *H*2. Meanwhile, *V*1 contains only *p*_1_ features with *n*1 samples and *V*2 has all *p*_1_+*p*_2_ features but with *n*_2_ samples. The linear ensemble of *V*1 and *V*2 is then our verticle stacking predictor *Vc*.

## Results

In this section, we first demonstrate the performance of *vertical stacking* and *horizontal stacking* of two RF components on a synthetic dataset and a real dataset. For both datasets we observe that *horizontal stacking* is not only more effective in reducing bias but also consistently outperforms its *vertical* counterpart in terms of MSE. Next, we demonstrate how linear stacking of different models operating on different non-overlaping features outperforms each individual component both in terms of bias and MSE reduction.

### Synthetic data

We generate a 2000×100 matrix of covariates drawn from a *Normal*(0,1) distribution. Then a random set of 100 weights are created, half are randomly selected to be "weak" predictors and are drawn from a *Uniform*(0,0.5) distribution while the other half are "strong" predictors drawn from *Uniform*(1.5,3). These weights are linearly combined with our covariates to create a set of 2000 responses. We then obtain the variance of the foregoing responses and add gaussian noise with a variance set at 3% the sample variance of the non-noisy data and add an intercept of 1.4. Finally, the noisy responses are normalized into the range of [−1,1].

Data is sectioned off into three groups. The first group is a set of 100 samples that constitutes our initial training set. The second group contains 50 samples and serves as a validation set for building the stacking ensemble predictors. The third group is 500 used for testing. The remaining samples are reserved for later addition into group one. The entire process is then repeated to generate 100 independent sets of data which are treated as replicates.

To illustrate the operation of *vertical* and *horizontal* stacking we divide our synthetic training data into the groups illustrated in Fig. 2 and build our individual and stacked models. Each individual model is a Random Forest with 50 trees and each tree utilizes one-third the input features. When splitting our features we make certain each horizontal group has at least 25 weak and 25 strong features. We then start adding samples, 20 at a time, to each group, remake our models and then re-estimate our MSE.

**Fig. 3:**
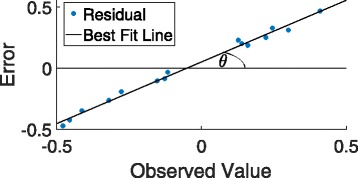
Example of the residuals from a biased estimator that shows the bias angle *θ* derived from best fit line

**Fig. 4:**
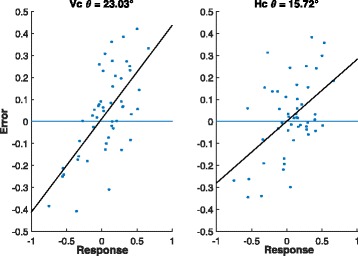
Residuals and best fit line for vertical stacking (left) and horizontal stacking (right) on synthetic data with 1200 training samples and 50 testing samples

*Bias analysis:* A plot of the residuals obtained from the RF estimates against the observed values often shows a linear trend [5] as against a random scatter about 0. Therefore, to assess the bias of the candidate model we can simply regress the residuals on the observed values. The angle (*θ*, see Fig. 3) the fitted line makes with the horizontal axis can be used as a measure of bias for that model. Larger values of *θ* indicates more bias and in the case of unbiasedness, *θ*=0. Figure 4 shows that the bias associated with *horizontal* stacking is substantially lower than that incurred in *vertical* stacking in accordance with the theoretical analysis of the previous section.

**Fig. 5:**
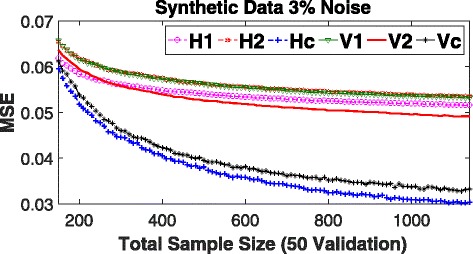
Mean Square Error (MSE) on Synthetic Data with increasingly large training sample sizes. *H*1, *H*2, *V*1, and *V*2 are individual RF models built using the template in Fig. 2. *Hc* and *Vc* are *horizontal* and *vertical* stacking models, respectively, built with their corresponding individual models. MSE shown is the average over 100 replicates

*MSE analysis:* Besides reduction in bias, we also argued in the previous section that the variance of *horizontal* stacking estimator should also be smaller than that of the *vertical* stacking estimator. Therefore, we would expect to see that the MSE associated with the predictions of the test samples would be consistently smaller for the former as compared to the latter. Figure 5 shows the performance of these two types on stacking in terms of prediction MSE for increasingly larger (training) sample sizes. Both *H*2 and *V*1 perform comparably across all the sample sizes under consideration. This is expected as both models have the same number of samples and features. *V*2, having all the features but fewer samples, overfits the data initially leading to higher prediction MSE as compared to *H*1, but as the sample size increases, *V*2 outperforms *H*1 to become the top single performer. However, regardless of the performances of individual components, the prediction MSE of *horizontal* stacking is consistently lower than that of the *vertical* stacking across all sample sizes under investigation.

### Analysis of CCLE data

**Fig. 6:**
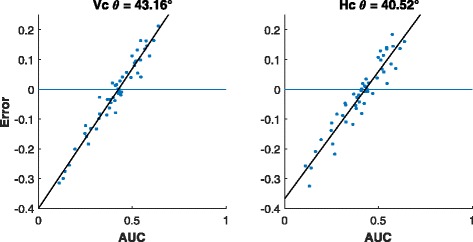
Residuals and best fit line for vertical stacking (left) and horizontal stacking (right) on 17-AAG data utilizing top 250 features 150 training samples and 50 testing samples

**Fig. 7:**
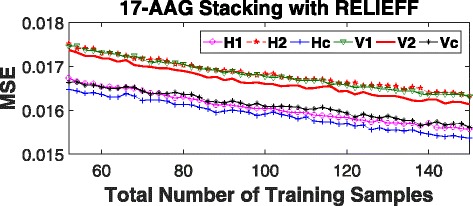
Mean square error (MSE) of AUC prediction on 17-AAG using top 250 gene expression features and 150 samples for validation. *H*1, *H*2, *V*1, and *V*2 are individual RF models built using the template in Fig. 2. *Hc* and *Vc* are *horizontal* and *vertical* stacking models, respectively, built with their corresponding individual models. MSE shown is the average over 100 iterations with new training/testing samples at each iteration

In order to test our theory on real dataset, we obtain gene expression and normalized Area Under the Curve (AUC) values for the cell lines tested on the drug 17-AAG from the Cancer Cell Line Encyclopedia (CCLE) [7]. From the available 19,000 gene expressions we use RELIEFF [20] to screen top 250 features. Similar to the synthetic case, we segregate 50 training samples into our vertical and horizontal groups, build individual predictive model RF with 50 trees, build the stacking model using a set of 150 samples, and obtain the prediction MSEs of candidate models on a set of 50 testing samples. We then add 2 training samples and reestimate the MSEs. We repeat this process until the training set has a total of 150 samples. The entire process is replicated 100 times with randomly selected training, testing, and validation samples in every iteration. The results are shown in Figs. 6 and 7. Similar to the synthetic results we see *horizontal* stacking consistently outperforming *vertical* stacking both in terms of reducing bias and prediction MSE.

### Analysis of GDSC data

We have access to four non-overlapping sources of data. (a) Drug sensitivity values for 265 compounds in the form of normalized AUC on 982 cancer cell lines supplied by GDSC [8] constitute the response variable. (b) Normalized gene expression for 982 cancer cell lines, also given by GDSC [8], forms one set of predictors. Once again, we use RELIEFF to screen the top 500 gene expression features from 18,000 available features. Since, the application of feature selection is drug dependent, the set of 500 selected features can be different for each drug. (c) Second set of predictors consists of the physical descriptors for 178 drug compounds calculated using the PaDEL software with the structure data files downloaded from PubChem [21]. The PaDEL defaults provide 1444 properties but we have removed features that had no variability across drugs resulting in 1181 chemical descriptors for each drug. Each chemical feature is a numerical value that can be continuous or discrete. (d) Finally, the last type of predictors contain information about the target proteins of 139 drugs under consideration. We generate the drug target predictor set by mining information from PubChem’s bioassay database [22]. For each drug, we look for either the reported *K*_*d*_ (dissociation constant) or *EC*_50_ (drug concentration required to reach 50% of maximal inhibition of the target) values for 419 kinases. In the case of multiple reported values, we first remove any major outliers using the following equation. Let $\vec {t_{i}}$ be a set of all reported *K*_*d*_ or *EC*_50_ values for target *i*. We then calculate the order of magnitude, $\vec {m}$ for each value and remove any values that deviates strongly from the most common order of magnitude as shown below.

12 $$\begin{array}{*{20}l} \vec{m} = \text{floor}\left(\log_{10}\left(\vec{t_{i}}\right)\right) \end{array} $$

13 $$\begin{array}{*{20}l} \text{Remove value if abs}\left(\vec{m}-\text{mode}\left(\tilde{\mathrm{m}}\right)\right) > 3 \end{array} $$

We have noticed this to be particularly effective when values are misreported as nanomolars instead of the standard micromolar. For the final value, we pick the median of all remaining values. These target values are then binarized using a threshold of one-half of the maximum dosage of the respective compound (taken from GDSC as well). A target is considered inhibited (value of 1) if the target value is less than one-half the max dose otherwise the value is set to 0.

**Fig. 8:**
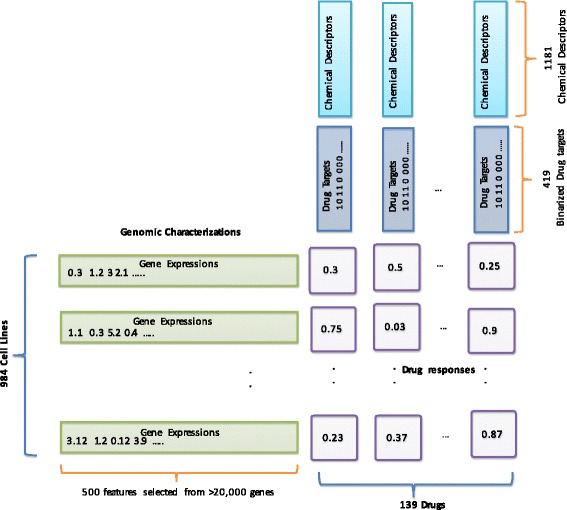
Pictorial Description of the data types used in our analysis

A graphical representation of various data sources and their relationships are shown in Fig. 8. The intersection of all data sets gives approximately 80000 samples. For model training and validation, the data was randomly split up into three sets. The first set contains approximately 60% of all samples and is used for training the individual predictors. The second set contains only 20% of the data and is used to estimate the stacking model parameters. Finally, the remaining 20% of the data is used for testing both individual and the ensemble prediction algorithms. A list of individual predictive models along with a short description for each of them is provided in Table 1. The “mean” model, where we simply utilize the mean AUC of each drug as our prediction, is included to establish a baseline. We measure the performance of individual candidate model using the Pearson correlation between all observed and predicted AUC values in our testing set as well as the mean squared error normalized with the mean square error of the mean predictor (NMSE). The results are shown in Table 2. In terms of individual performances, the genomic features outperformed the drug based features. Using genomic features, the RF produced a correlation of 0.7276 and NMSE of 0.7910 outperforming the remaining candidate models. We also note that modeling the centered AUC with drug target data works significantly better than modeling the raw AUCs. Consequently, we remove KNN Direct and NN Phy Direct from any further consideration.

**Table 1:** Explanation of all individual techniques used to predict drug AUC. Methods utilizing Residuals predict the sample-mean centered sensitivities (actual AUC- mean AUC) instead of the AUC directly

Method	Description
Mean	Prediction using the mean AUC of each drug
KNN Direct	K Nearest Neighbor (KNN) Approach using the actual AUC with drug target data
KNN Residual	KNN using the residuals with drug target data
NN GE	Neural Network on Gene Expressions
NN Phy Direct	Neural Network on Chemical Descriptors of drugs
RF Phy Residual	Random Forest on Chemical Descriptors of drugs using the residuals
RF GE	Random Forest on Gene expression

**Table 2:** Performance of Single Predictors in terms of correlation coefficient between predicted and actual AUCs (correlation) and normalized mean square error (NMSE) for predicting AUC. Models used for building higher order linear ensembles are shown in bold

Method	Correlation	NMSE
**Mean**	**0.6345**	**1**
**KNN Residual**	**0.6786**	**0.931**
KNN Direct	0.3623	1.555
**NN GE**	**0.7033**	**0.8613**
NN Phy Direct	0.3485	1.947
**RF Phy Residual**	**0.6819**	**0.8960**
**RF GE**	**0.7276**	**0.7910**

Next, we combine pairs of five surviving individual candidate models utilizing the linear stacking approach to obtain the second order linear ensemble. The performance of 10 second order linear ensembles, in terms of correlation and NMSE, are shown in Table 3. Observe that the linear ensemble of top two single models (RF GE and NN GE) does not deliver the best predictive performance in this setup. Instead, the top 3 performers (RF GE - KNN Residual, RF GE - RF Phys Residual, NN GE - KNN Residual) comprise of individual models that are trained on uncorrelated feature sets. In fact, linear combination of RF and NN, both trained on gene expression data performs worse than RF GE alone. A potential reason for this phenomenon is multicollinearity among the members in the linear ensemble. In either case it is plain to see that when stacking models it is best to include as much complementary information as possible.

**Table 3:** Correlation coefficients and NMSEs, in parenthesis, of second order linear ensemble with two component models for AUC prediction. Top 3 predictors are shown in bold

	KNN residual	NN GE	RF phy residual	RF GE
Mean	0.7181(0.8092)	0.7120(0.8266)	0.7090(0.8324)	0.7264(0.7919)
KNN Residual		**0.7492(0.7341)**	0.7197(0.8092)	**0.7550(0.7225)**
NN GE			0.7455(0.7457)	0.7258(0.7919)
RF Phys Residual				**0.7504(0.7341)**

Finally, all five individual predictive models are combined using linear stacking to obtain a single linear ensemble predictive model. The performance of this ensemble is reported in Table 4 along with the performance of the best single predictive model (RF GE) and the best second order linear ensemble (RF GE- KNN Residual). Observe that the final linear ensemble substantially outperforms the other two candidates both in terms of correlation and NMSE.

**Table 4:** Correlation coefficient between predicted and actual AUCs (correlation), and normalized mean square error (NMSE) for predicting AUC of our best single predictor, the *RF GE*, and linear stacking of five individual predictive models that appear in bold in Table 2

	Correlation	NMSE
RF GE	0.7276	0.791
RF GE + KNN Residual	0.7550	0.7225
Linear Stacking Ensemble	0.7746	0.6705

## Discussion

*Bias analysis:* Multiple methods have been proposed to correct the bias in Random Forests [5, 6]. Our foregoing theoretical discussion and simulation study suggest that appropriately designed linear stacking of individual predictive models is also an effective debiasing devise that can also improve prediction mean square errors. Here we explore how linear stacking compares with extant methodologies for bias correction in RF. In particular we use the **BC1** bias correction algorithm proposed in [6] where we fit a second RF with residuals as the response and the validation data as the covariates and then obtain the final prediction of AUC in the test set by adding the predicted residuals (obtained from the second RF) to the predicted values of the AUC generated by the principle RF fitted on the training data. Note that **BC1** outperforms the remaining 4 algorithms discussed in [6]. In the second method, **RRot**, we estimate the residuals using the same method as in **BC1**, however instead of adding the residuals back into our prediction we first rotate the residuals until the best fit regression line between residual and predicted values of the response is horizontal [5]. The results of these methods is shown in Table 5. Bootstrap confidence intervals are created using 1000 bootstrap samples. We see that the linear stacking ensemble has the best performance among all other approaches in terms of high correlation, low MSE and low bias. Averaging over all drugs, the linear stacking ensemble yields the lowest average *θ*, $\bar {\theta }$, of 34.25°as compared to that of the standard RF trained on gene expression data. The bias corrected version of RF infact increases $\bar {\theta }$ hence we do not investigate **BC1** and **RRot** any further.

**Table 5:** Comparison of Bias Correction techniques for improving bias angle and overall error. From top to bottom we have our best individual predictor RF GE (bolded). RF GE ensembled with our KNN utilizing the drug targets. RF GE ensembled with NN GE alone. RF GE ensembled with RF Phys Residual. Linear ensemble of all methods that are bolded in Table 2. For each method we shows correlation coefficient between predicted and actual AUCs (correlation), normalized mean squared error(NMSE), mean *θ* across all drugs (*θ*_*μ*_), and 95% bootstrap confidence interval lower and upper bounds, (*θ*_*L*_ and *θ*_*H*_ respectively) BC1 and RRot denote our RF GE corrected using techniques found in [5]

	Correlation	NMSE	*θ*_*μ*_	*θ*_*L*_	*θ*_*H*_
**RF GE**	**0.7276**	**0.7910**	**38.27**°	**37.34**°	**39.04**°
RF GE + KNN Residual	0.7550	0.7225	35.23°	34.07°	36.30°
RF GE + NNGE	0.7258	0.7919	38.22°	37.39°	39.03°
RF GE + RF Phys Residual	0.7504	0.7341	35.26°	34.23°	36.12°
Linear StackingEnsemble	0.7746	0.6705	34.25°	33.15°	35.26°
BC1	0.7184	0.8092	40.61°	40.00°	41.11°
RRot	0.7084	0.8382	40.60°	40.02°	41.12°

**Fig. 9:**
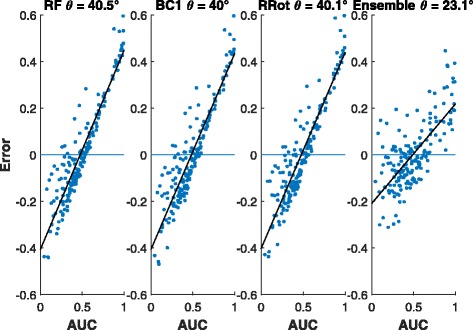
Residuals and bias angle from Belinostat AUC predictions. From left to right. Regular Random Forest (RF). BC1 RF. RRot RF. Linear ensemble from all data sources

**Fig. 10:**
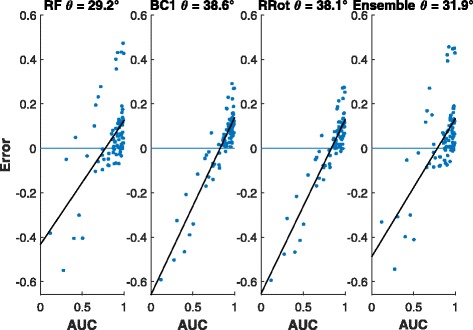
Residuals and bias angle from AZ628 AUC predictions. From left to right. Regular Random Forest (RF), BC1 RF, RRot RF, and Linear ensemble from all data sources

**Fig. 11:**
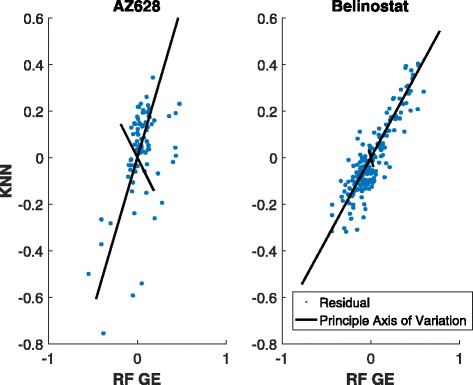
2D Residual Plot for Belinostat and AZ628 graphed with normalized principle axis of variation. X-axis shows the residual value for RF GE and y-axis is for the KNN Residual. Note that the principle axes in Belinostat is more heavily directed and has normalized eigenvalues of [0.95, 0.05] into a single direction than in AZ628 which has normalized eigenvalues of [0.76, 0.23] indicating potential improvement in ensemble classifier

To visually assess the effect of variance condition (*τ*^2^/*σ*^2^≫1) on the efficacy of linear stacking in reducing bias we offer the residual plots of two drugs Belinostat (Fig. 9) and AZ628 (Fig. 10). The former figure demonstrates that linear stacking efficiently reduces the bias while the latter shows a scenario where linear stacking is unable to correct the bias. To explain this phenomenon we generate the scatter plot of the residuals for our 2 best performing individual methods, the RF GE and KNN Residual, for these two drugs. The left panel of Fig. 11 shows this plot for AZ628 and the right panel corresponds to Belinostat. From these plots we see that the residuals for RF and KNN for Belinostat have a dominant principle axis of variation, with the normalized eigen values being 0.95 and 0.05. This satisfies the variance condition and hence we expect stacking operation to reduce bias substantially. However, for AZD628, the normalized eigen values are 0.76 and 0.23. Given that the ratio of these eigen values is close to 1, linear stacking is not guaranteed to reduce bias. Clearly, the variance condition offers an insight as to whether linear stacking will be beneficial or not. For higher (>2) order linear stacking, we recommend to perform an eigen analysis on the residuals after all the individual models are fitted. If the dominant eigen value explains >90*%* of the variation, linear stacking will efficiently correct for bias. A more detailed investigation of the eigen threshold is beyond the scope of this study but is certainly a subject of future explorations.

## Conclusions

Drug interactions of cancer cell lines are complex biological processes that can not be fully characterized using only genomic and drug properties. Accurate drug sensitivity predictions for personalized medicine will require the use of a variety of feature sets from multiple data sources. In this article we have shown that by incorporating drug target data from Pubchem and the physical properties generated using PaDEL we are able to improve the prediction accuracy of a Random Forest model trained on gene expression data. In particular, we have shown that such ensemble learners are effective in automatically removing the bias inherent in the Random Forest models. We have also derived a necessary condition for the linear ensemble to be an effective debiasing devising and described a degined approach to stacking operation. In the future other sources of data can be included to improve prediction accuracy. For example recent models built on protein-protein interaction networks [23] could provide information that is not captured by our current stacked model. However, we note that the entire theoretically premise is built upon the assumption of linear bias. We propose to investigate a more general stacking approach to handle non-linear biases.

## Abbreviations

AUC: Area under the curve
DL: Deep learning
KNN: k-Nearest neighbor
MSE: Mean squared error
NMSE: Normalized mean squared error
NN: Neural network
RF: Random forest
